# Investigation of Radiation Oncologists’ Awareness of Online Reputation Management

**DOI:** 10.2196/10530

**Published:** 2019-04-01

**Authors:** Jonathan Fredric Waxer, Sudesh Srivastav, Christian Steven DiBiase, Steven Joseph DiBiase

**Affiliations:** 1 Tulane University School of Medicine New Orleans, LA United States; 2 Department of Global Biostatistics and Data Science Tulane University School of Public Health and Tropical Medicine New Orleans, LA United States; 3 Webb School of Knoxville Knoxville, TN United States; 4 Department of Radiation Oncology New York-Presbyterian Queens Weill Cornell Medicine Flushing, NY United States

**Keywords:** reputation, management, internet, patient satisfaction, surveys and questionnaires, radiation oncology

## Abstract

**Background:**

Online reputation management (ORM) is an emerging practice strategy that emphasizes the systematic and proactive monitoring of online reviews relating to one’s professional reputation.

**Objective:**

We developed this survey project to assess whether radiation oncologists are aware of ORM and how it is utilized in their practices. We hypothesized that ORM is largely unknown by most practicing radiation oncologists and that little time is spent actively managing their reputations.

**Methods:**

An online survey was submitted to 1222 radiation oncologists using the Qualtrics research platform. Physician emails were gathered from the American Society for Radiation Oncology member directory. A total of 85 physicians initiated the survey, whereas 76 physicians completed more than or equal to 94% (15/16) of the survey questions and were subsequently used in our analyses. The survey consisted of 15 questions querying practice demographics, patient satisfaction determination, ORM understanding, and activities to address ORM and 1 question for physicians to opt-in to a US $50 Amazon gift card raffle. The survey data were summarized using a frequency table, and data were analyzed using the Chi-square test, Fisher exact test, and Spearman correlation coefficients.

**Results:**

We calculated a 7% (85/1222) response rate for our survey, with a completion rate of 89% (76/85). A majority of respondents (97%, 74/76) endorsed being somewhat or strongly concerned about patient satisfaction (*P*<.001). However, 58% (44/76) of respondents reported spending 0 hours per week reviewing or managing their online reputation and 39% (30/76) reported spending less than 1 hour per week (*P*<.001). A majority of physicians (58%, 44/76) endorsed no familiarity with ORM (*P*<.001) and 70% (53/76) did not actively manage their online reputation (*P*<.001). Although 83% (63/76) of respondents strongly or somewhat believed that patients read online reviews (*P*<.001), 57% (43/76) of respondents did not check their online reviews (*P*=.25) and 80% (61/76) endorsed never responding to online reviews (*P*<.001). Moreover, 58% (44/76) of the respondents strongly or somewhat supported the idea of managing their online reputation going forward (*P*=.001). In addition, 11 out of the 28 pairs of questions asked in our correlation studies reached statistical significance. Degree of concern for patient satisfaction and the notion of managing one’s ORM going forward were the 2 most frequently correlated topics of statistical significance in our analyses.

**Conclusions:**

ORM is presently under-recognized in radiation oncology. Although most practitioners are concerned about patient satisfaction, little effort is directed toward the internet on this matter. ORM offers an area of practice improvement for many practicing radiation oncologists.

## Introduction

### Theory

The path to becoming a physician involves a decade-long investment in time and money, making a physician’s professional reputation one of the most valuable parts of their practice. Online reputation management (ORM) has been a growing activity in the last decade. The idea of ORM is to systematically monitor, analyze, and filter online media sources and even interact with consumers via online reviews. In health care, ORM has been largely overlooked from a literature perspective, with limited articles dedicated to its presence, benefits, or practices. Despite the current paucity of literature regarding ORM in the health care setting, we believe the study of ORM is indicated, especially because of the numerous existing studies that discuss physician review websites (PRW) and how that form of data can guide future changes in practice. Furthermore, health maintenance organizations and other payers increasingly use patient satisfaction reports to profile individual physicians and guide physician compensation [1]. These examples highlight only a few examples of why physicians should be educated and up to date on this topic.

### Prior Work

As patients increasingly turn to the internet to search for health information and health care providers [2], online forums have become increasingly popular, and popular large-scale websites such as Yelp, Facebook, and Google Reviews now publish reviews on nearly every aspect of life. Other online forums, known as PRWs, have surfaced that solely discuss health care providers. PRWs are online services that allow patients and other third-party reviewers to grade physicians and hospitals in an online forum. Some examples of PRWs include Healthgrades.com, WebMD.com, ZocDoc.com, Vitals.com, and RateMDs.com.

Research on this topic has suggested that the popularity of PRWs is steadily increasing, and, as an example, the number of reviews on RateMDs.com has grown from 2475 reviews in 2005 to 112,024 in 2010 [3]. In 2012, 36% of surveyed Americans reported having searched for a physician on the internet [4], and over the past decade, the percentage of individuals that utilized the internet to obtain health information increased from 20% to 60% [3]. A survey of health care consumers in 2011 found that 28% (1120/4000) of respondents searched online for information regarding the quality of care provided by a primary care physician or a medical specialist, which was an increase from 24% in 2010 (960/4000). In addition, this number was found to be as high as 34% among younger generations [5], and based on a survey in 2015, more than a quarter of young parents selected a pediatrician for their child by using the internet [6]. This upward trend is expected to steadily increase as the ease of technological access improves and as the age demographics shift—resulting in a society of proportionally more tech-savvy individuals.

### Hypotheses

We developed this survey project to assess whether radiation oncologists are aware of ORM and how it is utilized in their practices. We hypothesized that ORM is largely unknown by most practicing radiation oncologists and that little time is spent actively managing their reputations.

## Methods

### Recruitment

Under institutional review board (IRB) guidelines, our anonymous survey project qualified as an exempt review. Collaborative Institutional Training Initiative certificates were completed and uploaded in the submission for all research personnel, and our study was subsequently approved by the IRB. A closed, voluntary online survey pertaining to ORM was created (Table 1).

The survey consisted of 16 questionnaire items over 2 pages that queried practice demographics, patient satisfaction, ORM understanding, and activities to address ORM. The survey questions were largely a collection of multiple-choice responses with a few fill-in-the-blank responses. Many of the questions utilized a 5-point Likert scale and asked respondents to rate their level of agreement with questions related to ORM: 1=strongly agree, 2=somewhat agree, 3=undecided, 4=somewhat disagree, and 5=strongly disagree [7].

Our target population was practicing radiation oncologists. We gathered 1222 radiation oncologists’ emails from the American Society for Radiation Oncology (ASTRO) membership database while excluding radiation physicists, nurses, and radiation oncology residents in training from our study. The electronic survey was created and subsequently delivered via email using the Qualtrics survey software. The electronic survey was tested for usability and technical functionality before being sent to our group of radiation oncologists. To ensure no duplicate entries were gathered, all respondents had a unique survey link, and users with the same internet protocol address were prevented from accessing the survey twice over the 3-month period in which the questionnaire was live. Electronic informed consent was delivered via email. Participants were told the purpose of the study, the investigator, the estimated length of time of the survey, and the IRB approval number. Informed consent was obtained by having the study participants begin the survey.

**Table 1:** Online reputation management physician survey and data. Overall, 16 questions were developed that assessed physician understanding of online reputation management, demographics, and opinions pertaining to patient satisfaction. For each multiple-choice response, frequency data, response percentages, and *P* values are included, where applicable.

Question		n (%)	*P* value
**1. Which of the following best describes your Radiation Oncology practice?**				
	A. Freestanding Practice	20 (26)	.02
	B. Hospital-Based Practice	26 (34)	.02
	C. Academic or University Practice	22 (29)	.02
	D. Other	8 (11)	.02
**2. How many years have you been practicing (since completing residency)?**				
	A. 0-5 years	20 (26)	.01
	B. 5-10 years	9 (12)	.01
	C. 10-15 years	13 (17)	.01
	D. 15-20 years	9 (12)	.01
	E. 20+ years	25(33)	.01
3. What state is your practice located in?				
**4. I am concerned about patient satisfaction.**				
	A. Strongly agree	58 (76)	<.001
	B. Somewhat agree	16 (21)	<.001
	C. Undecided	2 (3)	<.001
	D. Somewhat disagree	0	<.001
	E. Strongly disagree	0	<.001
**5. Does your practice perform patient satisfaction surveys?**				
	A. Yes, on paper	49 (65)	<.001
	B. Yes, online	20 (26)	<.001
	C. No	7 (9)	<.001
**6. I am familiar with the term Online Reputation Management (ORM).**				
	A. Yes	32 (42)	<.001
	B. No	44 (58)	<.001
**7. Which of the following best describes your level of management of your online reputation?**				
	A. I do not manage my online reputation	53 (70)	<.001
	B. I read online reviews	15 (20)	<.001
	C. I actively manage online reviews (ie, respond to negative/positive comments)	0	<.001
	D. I proactively ask patients to write and post reviews about their care online	3 (4)	<.001
	E. I pay someone to manage my online reputation	5 (6)	<.001
**8. How much time per week do you spend reviewing/managing your online reputation?**				
	A. <1 hour	30 (39)	<.001
	B. 1-2 hours	2 (3)	<.001
	C. 2-3 hours	0	<.001
	D. 3+ hours	0	<.001
	E. None at all	44 (58)	<.001
**9. I am concerned about my reputation.**				
	A. Strongly agree	47 (62)	<.001
	B. Somewhat agree	21 (28)	<.001
	C. Undecided	4 (5)	<.001
	D. Somewhat disagree	3 (4)	<.001
	E. Strongly disagree	1 (1)	<.001
**10. I am aware of third-party physician review sites.**				
	A. Strongly agree	33 (44)	<.001
	B. Somewhat agree	26 (34)	<.001
	C. Undecided	7 (9)	<.001
	D. Somewhat disagree	7 (9)	<.001
	E. Strongly disagree	3 (4)	<.001
**11. I believe online reviews are more impactful than ‘word of mouth.’**				
	A. Strongly agree	8 (11)	<.001
	B. Somewhat agree	22 (29)	<.001
	C. Undecided	26 (34)	<.001
	D. Somewhat disagree	13 (17)	<.001
	E. Strongly disagree	7 (9)	<.001
**12. I believe that patients read online reviews.**				
	A. Strongly agree	23 (30)	<.001
	B. Somewhat agree	40 (53)	<.001
	C. Undecided	8 (11)	<.001
	D. Somewhat disagree	4 (5)	<.001
	E. Strongly disagree	1 (1)	<.001
**13. I check online reviews that discuss my practice.**				
	A. Yes	33 (43)	.25
	B. No	43 (57)	.25
**14. I respond to online reviews that discuss my practice.**				
	A. Always	3 (4)	<.001
	B. Sometimes	12 (16)	<.001
	C. Never	61 (80)	<.001
**15. I welcome the idea of managing my online reputation.**				
	A. Strongly agree	20 (26)	.001
	B. Somewhat agree	24 (32)	.001
	C. Undecided	18 (24)	.001
	D. Somewhat disagree	11 (14)	.001
	E. Strongly disagree	3 (4)	.001
16. Please provide your email address below if you wish to be entered into the drawing to win one of five US $50 Amazon gift cards.				

Participants were given 3 months to complete the survey, and 4 email announcements were sent as reminders from December 2016 to February 2017 to participants who had not previously completed the survey as the study deadline approached. Respondents were able to review and change their answers before survey submission, and a completeness check tool was not utilized. We used 5 $50 Amazon gift card raffles as incentives to improve participation. The survey data were automatically captured in Qualtrics. At the completion of our data gathering stage, the Qualtrics survey data were imported into Microsoft Excel, deidentified, and summarized using a frequency table listing frequency, percentages, and *P* values (Table 1).

### Statistical Analysis

All data relating to study specific aims were summarized using descriptive statistics. Frequency tables were drawn up for nominal and ordinal data. The Chi-square and Fisher exact test methods were applied to compare association and proportions. The Chi-square test was used because of the varying degrees of freedom per question and would be able to indicate how likely our observed distribution was because of chance. *P* values for statistical significance were then analyzed using a 2-sided 5% significance level throughout the analyses. Correlation studies were conducted using the Spearman correlation coefficient. Correlation coefficients were characterized as either weak (r<.30), moderate (.30≤r≤.70), or strong (r>.70). We performed our analyses on our multiple-choice, demographic-defining questions (1 and 2) and our survey questions that contained a 5-point Likert scale: 4, 9, 10, 11, 12, and 15 (Table 2). All data analyses, summaries, and listing were performed using SAS software (version 9 or higher in a Windows environment).

## Results

Of the 1222 invites, 85 surveys were initiated, 79 were submitted, and 76 had answered more than or equal to 94% (15/16) of the total survey questions and were subsequently included in our analyses. A completeness rate of 94% was utilized as no completeness check was enforced, and the final question indicated whether participants wished to be entered in our gift card raffle and was not to be included in our analysis. A completion rate of 89% (76/85) was calculated, and we calculated our response rate at 7% (85/1222). We received responses from 28 separate states, with the highest concentration of respondents in the Northeast and Southern United States. In addition, 1 survey participant engaged in locum tenens, and 2 did not specify their location (see Multimedia Appendix 1). Overall, 26% (20/76) of our respondents were involved in free-standing practices, totaling fewer percentages than either hospital-based (26/76, 34%), academic/university-based practices (22/76, 29%), or other (8/76, 11%). When queried about the importance of patient satisfaction, a majority of respondents (74/76, 97%) endorsed being somewhat or strongly concerned about patient satisfaction (*P*<.001), as evident by the 91% (69/76) of respondents that reported conducting either paper or online surveys in their respective practices (*P*<.001).

When describing ORM, a majority of physicians (43/76, 57%) endorsed no familiarity with this practice management activity (*P*<.001) and 70% (53/76) did not actively manage their online reputation (*P<*.001). Although 83% (63/76) of respondents strongly or somewhat believed that patients read online reviews (*P<*.001), 57% (43/76) of respondents did not check their online reviews (*P=*.25) and 80% (61/76) endorsed never responding to online reviews (*P<*.001). However, when it came to the amount of time spent per week reviewing or managing their online reputation, 58% (44/76) of respondents reported spending 0 hours per week and 39% (30/76) reported spending less than 1 hour per week (*P<*.001). In terms of an area of active practice improvement, 58% (44/76) of respondents strongly or somewhat supported the idea of managing their online reputation going forward (*P=*.0012).

Overall, 11 out of the 28 pairs of questions asked in our correlation studies reached statistical significance (Table 2). The degree of concern for patient satisfaction and the notion of managing one’s ORM going forward were the 2 most frequently correlated topics of statistical significance in our analyses. Our strongest correlation was observed between a respondent’s belief that online reviews are more impactful than word of mouth (Q11) and their belief that patients read online reviews (Q12; *r*=.46, *P*<.001). Other statistically significant positive correlations of moderate strength occurred between a radiation oncologist’s type of practice (Q1) and their degree of concern for their reputation (Q9; r=.30, *P*=.008); their type of practice (Q1) and their degree of agreement that patients read online reviews (Q12; *r*=.37, *P*=.001); and their type of practice (Q1) and the notion of managing their online reputation going forward (Q15; *r*=.33, *P*=.003). 

There were other statistically significant positive correlations of weak strength between a respondents degree of concern for patient satisfaction (Q4) and their reported awareness of third-party PRWs (Q10; *r*=.23, *P*=.04); their degree of concern for patient satisfaction (Q4) and their belief that online reviews are more impactful than word of mouth (Q11; *r*=.26, *P*=.28); and their degree of concern for patient satisfaction (Q4) and the notion of managing their online reputation going forward (Q15); (*r*=.28, *P*=.01).

**Table 2:** Spearman correlation coefficients (N=76). Correlation studies of our multiple-choice, demographic-defining questions (1 and 2) and our multiple-choice questions utilizing Likert scales (4, 9, 10, 11, 12, and 15) were conducted using the Spearman correlation coefficient. Statistically significant correlations are shown in italics. For each pair of questions, correlation coefficients and *P* values are included.

Question (Q) number	Q 1	Q2	Q4	Q9	Q10	Q11	Q12	Q15
Q1	1	*r*=−.03, *P*=.78	*r=.23, *P*=.04*	*r*=.19, *P*=.10	*r*=.02, *P*=.86	*r*=.04, *P*=.76	*r*=−.05, *P*=.64	*r=.30, *P*=.008*
Q2	*r*=−.03, *P*=.78	1	*r*=−.07, *P*=.57	*r*=.12, *P*=.28	*r*=−.14, *P*=.21	*r=.26, *P*=.03*	*r*=.18, *P*=.12	*r*=−.14, *P*=.22
Q4	*r=.23, *P*=.04*	*r*=−.07, *P*=.57	1	*r=.29, *P*=.01*	*r=.27, *P*=.02*	*r*=.06, *P*=.62	*r*=.16, *P*=.18	*r=.23, *P*=.04*
Q9	*r*=.19, *P*=.10	*r*=.12, *P*=.28	*r=.29, *P*=.01*	1	*r*=.21, *P*=.07	*r*=.20, *P*=.08	*r*=.20, *P*=.08	*r=.37, *P*=.001*
Q10	*r*=.02, *P*=.86	*r*=−.14, *P*=.21	*r=.27, *P*=.02*	*r*=.21, *P*=.07	1	*r*=.02, *P*=.84	*r*=.13, *P*=.26	*r=.26, *P*=.02*
Q11	*r*=.04, *P*=.76	*r=.26, *P*=.03*	*r*=.06, *P*=.62	*r*=.20, *P*=.08	*r*=.02, *P*=.84	1	*r=.46, P≤.001*	*r=.28, *P*=.01*
Q12	*r*=−.05, *P*=.64	*r*=.18, *P*=.12	*r*=.16, *P*=.18	*r*=.20, *P*=.08	*r*=.13, *P*=.26	*r=.46, P≤.001*	1	*r=.34, *P*=.003*
Q15	*r=.30, *P*=.008*	*r*=−.14, *P*=.22	*r=.23, *P*=.04*	*r=.37, *P*=.001*	*r=.26, *P*=.02*	*r=.28, *P*=.01*	*r=.34, *P*=.003*	1

 Additional statistically significant positive correlations of weak strength were observed between one’s type of practice (Q1) and their degree of concern for their reputation (Q9; *r*=.23, *P*=.04); one’s type of practice (Q1) and their reported awareness of third-party PRWs (Q10; *r*=.29, *P*=.01); and one’s type of practice (Q1) and their degree of concern for patient satisfaction (Q4; *r*=.27, *P*=.02). Finally, a statistically significant positive correlation of weak strength was observed between a radiation oncologist’s number of years since completing residency (Q2) and their belief that online reviews are more impactful than word of mouth (Q11; *r*=.26, *P*=.03).

## Discussion

### Principal Findings

To our knowledge, this is the first study assessing the ORM of practicing radiation oncologists in the scientific literature. Radiation oncology, as a specialty, is dependent upon referrals, and therefore, we hoped to educate practicing radiation oncologists on the importance of managing their online reputation and to provide future strategies to increase overall patient satisfaction, retention, and referral. Our results indicate that radiation oncologists are very concerned about their professional reputation and patient satisfaction regardless of their type of practice; however, very little time is spent actively managing their online reputation as a majority of respondents (69/76, 91%) already utilize paper or online surveys in their practice, but so few physicians reported spending any meaningful amount of time actively managing their online reputation. Furthermore, concern for patient satisfaction and the notion of managing one’s ORM going forward were the 2 most frequently correlated topics of statistical significance in our survey. We also observed that a radiation oncologist’s degree of concern for patient satisfaction and their degree of agreement in managing their ORM in the future were correlated with those who identified working within free-standing practices versus hospital or academic/university-based practices. In addition, the belief that online reviews are more impactful than *word of mouth* was correlated with radiation oncologists that had fewer years since completing residency. These findings support the notion that ORM is an emerging area of practice management that is presently under-recognized in radiation oncology but offers a meaningful avenue for practice improvement and is of increased interest among younger radiation oncologists or those that operate in free-standing practices.

### Comparison With Prior Work

How might ORM be relevant to health care practitioners? In a study, Fox and Jones showed that 61% of American adults look toward the internet for health information, and that percentage is theorized to be growing as ease of access to technology increases and younger generations transition into adulthood [8]. A separate study performed by the *Journal of the American Medical Association* reported that 25% of US adults consulted online physician rating sites, and more than 33% of online viewers went to a physician or avoided one based on their ratings [9]. Furthermore, a recent study analyzed online Healthgrades reviews of 2679 radiation oncologists and found that their “likelihood to recommend to family and friends” score was significantly lower for physicians with fewer numbers of online reviews (<10) compared with colleagues with more than 10 reviews [10]. These are just a few examples that underscore the use of online health information and how public information might influence prospective patients. As alluded to before, much literature has been written on patient satisfaction, but despite the increased accessibility of these data, Rider and Perrin showed that less than 25% of primary care physicians used these data for improving patient care and even fewer report using the information to change their practice [11].

Prabhu et al [12] looked at the top 10 Google search results for 4443 Medicare-practicing radiation oncologists in the United States and Puerto Rico. These search results were extracted, categorized, and reviewed. They found that physician-, hospital-, and health care–controlled websites (39.3%) and third-party websites (25.7%) were the 2 most observed domain types. However, social media and academic journal articles accounted for only 6.7% and 3.4% of the results, respectively. They identified that self-controlled online content, such as social media websites, was disproportionately lacking, and they went on to discuss potential proactive strategies [12].

Many proactive strategies that can improve a physician’s online presence exist with a minimal or modest additional time investment. The overarching goal of these efforts is to have better awareness and control of the published online content as well as a physician’s search engine rankings [12]. For example, as surveys are already implemented at most of our respondents’ practices, a proactive approach includes asking all patients to consider completing an end-visit survey online. The surveys could also provide an opportunity for patients to write testimonials, and they could be given the option to have their testimonials published online. These testimonials can be easily published by creating a personal blog or Web page that can further share patient education materials as well as one’s personal and clinical research interests. Other strategies suggested by Prabhu et al [12] include having each provider go to the many existing PRWs (Healthgrades, RateMDs.com, ZocDoc.com, etc) and edit their listed contact information for accuracy as well as utilizing professional social networking sites, such as Linkedin.com or Doximity.com, that reflect their curriculum vitae. Furthermore, in a study from Saudi Arabia, Househ showed that 99% of doctors utilize social media for personal use, but only 65% of doctors utilize social media for professional use [13]. The various social media apps can serve as a more personable and flexible platform to interact directly with patients and for increasing a physician’s online visibility and transparency. It can also provide the opportunity to fully control and customize one’s public information, including biographical data, that may otherwise be limited by official hospital or health care system websites.

King et al [14] used a mixed-methods approach in the United Kingdom to investigate the most important factors patients considered when choosing to see a health provider. By analyzing the relevant literature and conducting survey questionnaires and focus groups, they found that information about hospital staff—mainly their competency level—was important to patients. Relevant information that was highlighted included the amount of experience, qualifications, place of education, and interpersonal skills. Furthermore, staff competence seemed to best be captured by past users’ reviews, and patients were willing to travel for higher ratings in this category. Other categories that were highlighted included information about medical facilities, such as the modernity of the facilities and their technological equipment, as well as hospital statistics. Information about how to get to the hospital was not found to be an important factor [14].

Unfavorable reviews are unavoidable in medical practices, especially because of the expansion and increased popularity of PRWs. Furthermore, physicians should understand the permanency of the internet. Even if certain posts are deleted, there are sites that keep records of deleted posts, pages, and message boards. By proactively surveying and publishing patient testimonials, monitoring and updating contact information for PRWs, and creating other social media platforms, a physician’s online reputation can be better controlled and shielded to look more well-rounded and less polarized—interspersing the few inevitable negative comments with many other neutral or positive responses.

In some cases, physicians may seek professional assistance. There has been a steady increase in the market demand of consultant companies offering expertise for these reputation services. Some notable labors that these ORM consultants might implement include conventional public relation activities, search engine reputation management, and building blogs and other social media channels for positive reviews. Our hope was to pique interest and awareness into the realm of ORM and help educate fellow radiation oncologists about the benefits of proactively managing their online reputation.

### Limitations

Due to the nature of being an electronic survey, selection bias is an important limitation of this study, wherein the participants who chose to respond may not be generalizable to the greater population of all practicing radiation oncologists. This lack of generalizability is further complicated by radiation oncology as a field. For example, Lewis et al showed that 48% of radiation oncologists practiced in nonacademic, radiation oncology–only private practices; 20% in academic practice; 14% in nonacademic, multispecialty practices; and 11% in solo practice [15]. However, the range of demographics recorded by our study participants suggests a more evenly distributed sample. Our diversity in physician demographics may suggest the applicability and relevance of this topic to a variety of professional settings in radiation oncology and provides some reassurance of the validity of our findings. Although an argument could be made that ORM most financially impacts physicians involved in free-standing practices, our survey respondents involved in free-standing practices (26%) totaled smaller percentages than either hospital-based (34%) or academic/university-based practices (29%). This finding suggests the overall interest and applicability of ORM was recognized by most radiation oncologists in varying types of practices.

Another limitation of our study is our low response rate of 7%. This was well below the average response rate of 16% for the ASTRO annual membership survey from 2017 [16], and that study did not provide any form of compensation for survey completion. An explanation for our below-average response rate could be because of a phenomenon called nonresponse bias—where a distinct difference exists between those who respond to a given survey and those who do not. For example, radiation oncologists that have some familiarity with ORM may feel more comfortable and confident in completing our survey, even if the survey is anonymous. If nonresponse bias did, in fact, account for our significantly lower response rate, then that would help explain why 47% our respondents were already familiar with ORM before survey completion, as that was a much higher percentage than we were expecting to observe. Eliminating this bias would, therefore, further strengthen our hypothesis that most radiation oncologists are not familiar with ORM and do not engage in regular practices catered toward strengthening their online reputation.

A final limitation of our survey was that the study had no objective testing component. By implementing a self-assessment of personal knowledge and practices, physicians may overestimate their perceived awareness or level of involvement in ORM. Future research utilizing direct observation would provide more objective data and insight regarding ORM and daily practices. Despite these limitations, we believe that the study is clinically meaningful and helps highlight underlying knowledge gaps in ORM. This underscoring can help direct educational efforts in the future. We believe more time should be allocated toward patient satisfaction and managing one’s online reputation as both the patient and the physician will benefit.

### Conclusions

The internet continues to exert profound effects on professional reputations in medical practices; patient satisfaction is increasingly becoming a metric to which physicians are rated and has already influenced physician compensation. This study indicates that a large majority of radiation oncologists are somewhat or strongly concerned about patient satisfaction, yet most were not familiar with ORM nor did they actively manage their online reputation. Furthermore, the concern for patient satisfaction and the notion of managing one’s ORM going forward were the 2 most frequently correlated topics in our survey. We also observed correlations between radiation oncologists with fewer years since completing residency and the belief that online reviews are more impactful than *word of mouth* as well as between those working within free-standing practices and the notion of managing their ORM in the future. It is important to understand the current attitudes surrounding one’s online reputation as well as the evolving role that PRWs and social media websites can have on patient referral and satisfaction. Many posts on social media can remain on the internet indefinitely, and just a few negative reviews can significantly impact a physician’s reputation and be enough to deter potential patients. Our goal was to help identify gaps in radiation oncologists’ understanding of ORM in hopes to raise awareness and persuade radiation oncologists to consider having a more active role in their online presence.

## Appendix

Multimedia Appendix 1 Geographic distribution map of the United States showing location of survey participants’ state of practice.

## Abbreviations

ASTRO: American Society for Radiation Oncology
IRB: institutional review board
ORM: online reputation management
PRW: physician review website
