# Supplementary material for: Investigation of Radiation Oncologists’ Awareness of Online Reputation Management
Source: JMIR Cancer. 2019 Apr 1;5(1):e10530. doi: 10.2196/10530 (PMC6462885; doi:10.2196/10530)
Supplement: Multimedia Appendix 1 [file cancer_v5i1e10530_app1.PPTX]

## Slide 1
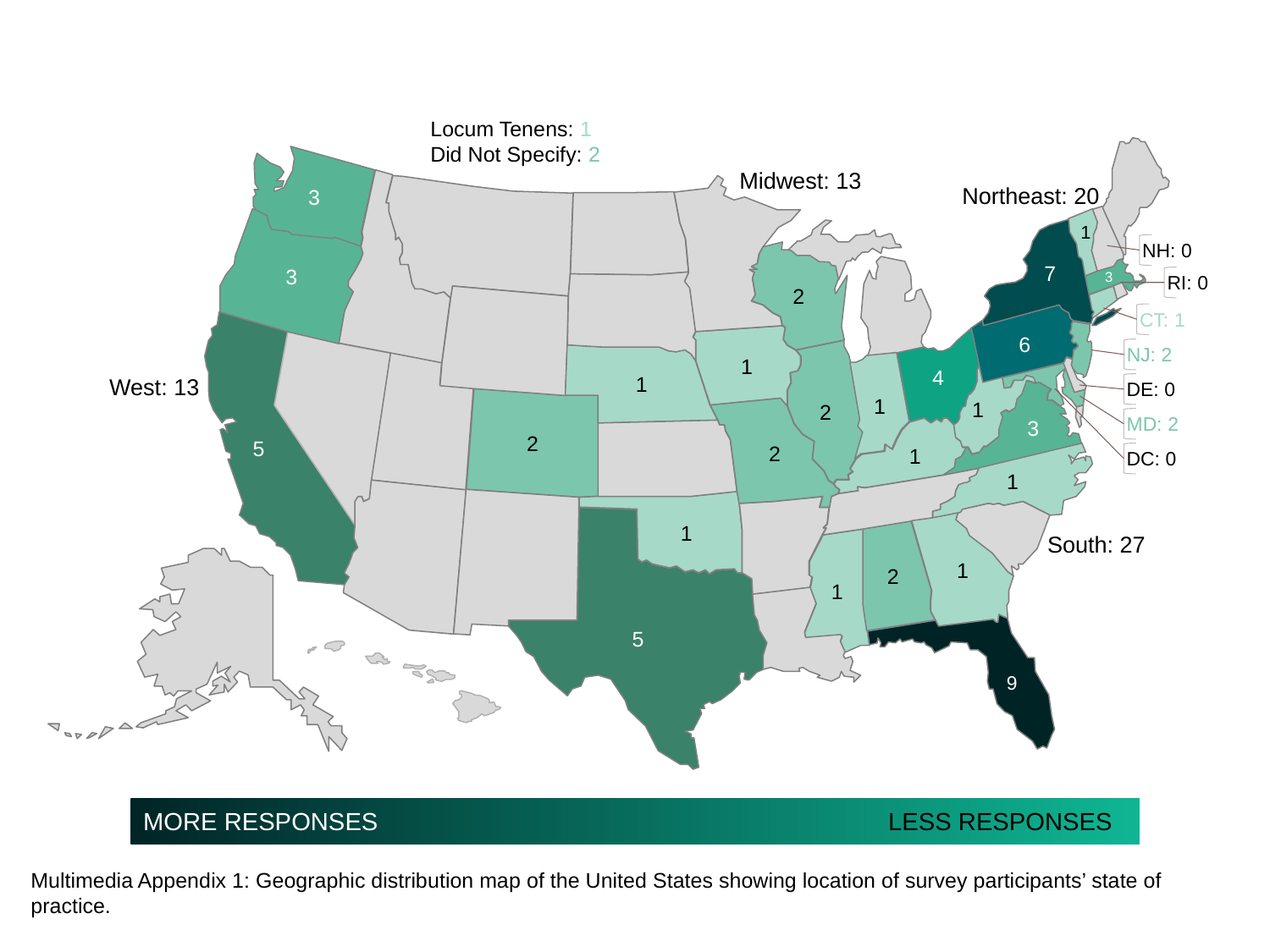

Locum Tenens: 1
Did Not Specify: 2
3
Midwest: 13
Northeast: 20
3
1
 7
NH: 0
2
3
RI: 0
CT: 1
6
 5
1
4
NJ: 2
2
1
1
 1
West: 13
DE: 0
 3
2
2
MD: 2
 1
1
DC: 0
 1
5
1
 2
South: 27
1
 9
LESS RESPONSES
MORE RESPONSES
Multimedia Appendix 1: Geographic distribution map of the United States showing location of survey participants’ state of practice.
